# Post-hip fracture rehabilitation outcomes of diabetic and non-diabetic elderly patients

**DOI:** 10.1080/07853890.2021.2009555

**Published:** 2021-12-03

**Authors:** Ronen Ben-Joseph, Barak Luboshitz, Rachel Heffez Ayzenfeld, Orit Twito

**Affiliations:** a Department of Geriatric Rehabilitation, Meir Medical Center, Kfar Saba, Israel; b Sackler Faculty of Medicine, Tel Aviv University, Tel Aviv, Israel; c Institute of Endocrinology, Meir Medical Center, Kfar Saba, Israel

**Keywords:** Hip fractures, rehabilitation, diabetes mellitus, osteoporosis, functional independence

## Abstract

**Background:**

Although patients with diabetes mellitus (DM) are at higher risk of hip fracture, data regarding the effect of DM on rehabilitation outcomes are limited.

**Methods:**

A retrospective single-centre study was conducted comparing elderly diabetic and non-diabetic patients with recent hip fracture, admitted to geriatric rehabilitation, 2014–2019. The functional independence measure (FIM) was used to assess physical and cognitive function. Delta-FIM was calculated by subtracting admission FIM from discharge FIM. One-year mortality, hospitalizations and fractures were assessed.

**Results:**

Six-hundred-thirty elderly patients, post-hip fracture were included, mean age 83 ± 7 years, 70.5% (444) women. Among them, 193 (30.6%) had type 2 DM, HbA1c 6.6 ± 1.25%. They were younger (81.4 *vs.* 84.3 years, *p* < .01) and had more co-morbidities including hypertension, chronic kidney disease, ischaemic heart disease and cerebrovascular disease. Baseline cognitive and motor scores were similar between groups. Delta motor-FIM was similar between diabetics and non-diabetics (15.56 ± 8.95 and 14.78 ± 8.79, respectively, *p* = .35). Multivariate regression analysis showed motor-FIM improvement was associated with higher BMI, male sex, and younger age, but not with DM. Cognitive FIM did not change significantly during rehabilitation in either group. Similar rates of patients were discharged to nursing care facilities. There was no difference in 1-year hospitalization or fracture rates. One-year, all-cause mortality was higher among diabetic patients (10.9 *vs.* 6.6%, respectively, *p* = .07). After adjusting for covariates, DM was associated with higher mortality risk (odds ratio = 2.78, 95% CI [1.28, 6.04], *p* = .01).

**Conclusions:**

Patients with well-controlled DM have similar post-hip fracture rehabilitation potential compared with non-diabetics, despite more co-morbidities. These results support resource allocation for post-hip fracture rehabilitation among patients with DM. The higher 1-year all-cause mortality in patients with DM reinforces the need for close follow-up and control of co-morbidities in this population.

## Introduction

Worldwide, hip fractures affect millions of elderly people each year, and are ranked among the top 10 causes of disability [1]. Global estimations predict that hip fractures will affect approximately 6% of men and 18% of women. The global number of hip fractures is increasing annually and is expected to reach 4.5 million by 2050, due to rapid population ageing and increased longevity [2,3]. Functional decline and diminished quality of life are common even after timely surgical management, with 10% of patients bedridden, 15% in long-term care facilities, and 80% using a walking aid 1 year after the hip fracture [4,5]. The direct costs associated with the estimated increased incidence of hip fractures are enormous because of the long period of hospitalization and subsequent rehabilitation [2].

Patients with either type 1 or type 2 diabetes mellitus (DM) have greater risk of osteoporosis and fractures, compared to non-diabetics. Notably, patients with diabetes are at significantly higher risk for hip fracture [6]. The risk for fragility fractures increases with duration of DM and is correlated with poor glycaemic control [7]. The underlying mechanism for bone fragility in DM is complex and includes low bone turnover, aberration of hormonal signalling and response, and changes in bone properties due to advanced glycation end products [8–10]. In addition, morbidity and mortality after hip fracture repair are higher among diabetic patients [11–13].

Data regarding the effect of DM on rehabilitation outcomes after hip fracture repair are limited and inconclusive. Several studies have shown that diabetic patients require longer rehabilitation following hip fracture repair [14] and achieve less successful rehabilitation results compared with non-diabetic patients [15,16]. Conversely, other studies showed similar rehabilitation outcomes between diabetic and non-diabetic patients [13,17]. A previous study showed no association between glycaemic control and rehabilitation outcomes or mortality of elderly diabetic patients after hip fracture repair [18].

This study compared clinical and functional characteristics of elderly diabetic and non-diabetic patients after hip fracture, and assessed the effect of DM on functional rehabilitation outcomes and 1-year prognosis.

## Methods

This single-centre, retrospective study evaluated all patients with a recent hip fracture who were admitted to a geriatric rehabilitation department during a 5-year period (2014–2019). The rehabilitation facility of Meir Medical Centre is in central Israel and serves a population of about one million people. All patients were at least 65 years old and were referred to the rehabilitation department several days after surgery.

Data were collected retrospectively from the electronic medical records of the medical centre. According to Israeli regulations, all patients after hip fracture, are eligible for rehabilitation, in an ambulatory or rehabilitation facility setting. The decision between the two options is made by the medical personnel, the patient and his/her family. Although medical parameters are a factor in this decision, glycaemic control is not one of them. During the study period, 2097 elderly patients underwent surgical treatment for proximal hip fracture at Meir Medical Centre, of whom 690 were referred to the inpatient geriatric rehabilitation centre. Information on patients who were not admitted to the rehabilitation facility was not available. After review, 60 patients were omitted from analyses, either because of a lack of functional outcome measurements or early termination of rehabilitation due to a medical condition. Ultimately, 630 patients were included in the cohort, of which 193 (30.6%) had diabetes. The diagnoses of DM were verified using the American Diabetes Association standard cut-offs based on pre-admission HbA1c and fasting glucose levels [19]. The study was approved by the Ethics Committee of Meir Medical Centre (0052-19-MMC) and met the standards of the Declaration of Helsinki. In accordance with Helsinki regulations for clinical studies based on chart review, informed consent was waived.

The Geriatric Rehabilitation Department at Meir Medical Centre is a post-acute inpatient rehabilitation facility with a multidisciplinary team of physicians, nurses, physical therapists, occupational therapists, speech therapists, dieticians and social workers. Individual interventions are delivered at a frequency depending on patient needs (Appendix 1). Rehabilitation care included: (1) 30–60 min daily of individual physical therapy (i.e. improving transferring, walking the length of a room, climbing stairs, equilibrium, muscle strength and joint range of motion [ROM]); (2) 30–60 min daily of individual occupational therapy (basic and instrumental activities of daily living, cognitive evaluation and stimulation, safety education); (3) 60 min daily of group exercise targeted to improve muscle strength, joint flexibility and ROM; and (4) walking with a physiotherapy aide according to the patient’s needs. A multidisciplinary team conference, coordinated by a geriatric specialist, is held within 5 d of admission and weekly thereafter to set individual goals of rehabilitation, review progress and discuss appropriate time for discharge.

The functional independence measure (FIM) was used to measure the patients’ level of disability on admission and at discharge. The FIM is an 18-item measurement tool that explores motor (physical) and cognitive function (Appendix 2). The motor subscale is composed of 13 items (eating, grooming, bathing, dressing of upper and lower body, toileting, bladder and bowel management, transferring between bed and chair, transferring in toilet and shower, walking and climbing stairs), and a cognitive subscale of 5 items (comprehension, expression, social interaction, problem solving and memory). Each item is scored on a 7-point ordinal scale, ranging from 1 (“total assistance with helper”) to 7 (“complete independence with no helper”). The higher the score, the more independent the patient is in performing the task associated with that item. The FIM is the most widely used assessment method in geriatric rehabilitation programmes and has proven inter-rater reliability and validity [20]. All FIM scores in the rehabilitation department were recorded by a geriatric specialist in consultation with the multidisciplinary team.

Demographic and clinical indices evaluated at admission included age, sex, body mass index (BMI), smoking status, comorbidities (hypertension, ischaemic heart disease, cerebrovascular disease, chronic kidney disease, obesity, dyslipidaemia and pressure sores), laboratory results (fasting glucose, creatinine, total cholesterol), cognitive and functional status [Mini-Mental State Examination (MMSE) score, and cognitive and motor FIM scores]. For patients with diabetes, the most recent pre-admission HbA1c level, spot urine albumin/creatinine ratio, and anti-DM drug therapy during rehabilitation (insulin and non-insulin) were retrieved. Functional and cognitive data at discharge from rehabilitation included discharge destination (home *vs.* nursing care), and motor FIM score. Delta motor FIM was calculated by subtracting admission from discharge scores. Long-term outcomes included recurrent fractures, hospitalizations and mortality within 1 year after discharge.

The data were analysed using SPSS software version 25 (SPSS Inc., Chicago, IL). Descriptive statistics were produced using frequencies for categorical variables, and means and standard deviations for continuous variables. The differences between the diabetic and non-diabetic patients regarding demographics, clinical characteristics, and rehabilitation outcomes were assessed using Chi-square tests for the discrete variables, and Mann–Whitney tests for continuous variables. Linear and logistic regression analyses were conducted to assess significant predictors for rehabilitation outcomes, among the demographic and clinical characteristics. The regressions were conducted for the whole sample, and separately for patients with diabetes. The level of significance (*p* value) was set at 5%.

## Results

A total of 630 elderly patients who were admitted for rehabilitation following a recent hip fracture matched the inclusion criteria. Their mean age was 83 ± 7 years, and 70.5% (444) were women. Patients with DM comprised 30.6% (193) of the sample and all were diagnosed with type 2 DM. Table 1 presents baseline characteristics of the diabetic and non-diabetic patients. Diabetic patients were younger than non-diabetics (81.4 ± 7.6 *vs.* 84.3 ± 7.1 years, respectively, *p* < .01) and had higher BMI (26.33 ± 4.81 *vs.* 24.69 ± 4.23 kg/m^2^, respectively, *p* < .01). Patients with DM had more co-morbidities, as well as higher creatinine levels in comparison to those without diabetes (1.06 ± 0.48 *vs.* 0.99 ± 0.47 mg/dl, respectively, *p* = .01), but lower total cholesterol levels (159.98 ± 38.55 *vs.* 168.40 ± 35.19 mg/dl, respectively, *p* value < .01), most likely due to more intensive use of lipid-lowering medications (48.7 *vs.* 33.9%, respectively, *p* < .01). Baseline cognitive and motor functions, as assessed by the MMSE, cognitive and motor FIM scores, were similar between the groups.

**Table 1.** Baseline characteristics of elderly patients with or without diabetes admitted to rehabilitation after hip fracture.

Variable	DM	Non-DM	*p* Value
*N* (%)	193 (30.6)	437 (69.4)	
Age, years	81.4 ± 7.6	84.3 ± 7.1	**< .01**
Sex			.85
Female, *n* (%)	135 (69.9)	309 (70.7)	
Male, *n* (%)	58 (30.1)	128 (29.3)	
Rehabilitation duration, days	29.2 ± 9.9	28.6 ± 9.4	.46
Clinical data at admission for rehabilitation			
BMI, kg/m^2^	26.33 ± 4.81	24.69 ± 4.34	**< .01**
Smoking, *n* (%)	28 (14.5)	48 (11.0)	.21
Hypertension, *n* (%)	161 (83.4)	313 (71.6)	**< .01**
Ischaemic heart disease, *n* (%)	54 (28.0)	78 (17.8)	**< .01**
Cerebrovascular disease, *n* (%)	63 (32.6)	98 (22.4)	**< .01**
Chronic kidney disease, *n* (%)	70 (36.3)	108 (24.7)	**< .01**
Obesity, *n* (%)	36 (18.7)	49 (11.2)	**.01**
Dyslipidaemia, *n* (%)	126 (65.3)	200 (45.8)	**< .01**
Use of lipid-lowering medications, *n* (%)	94 (48.7)	148 (33.9)	**<.01**
Pressure sores, *n* (%)	28 (14.5)	40 (9.2)	.046
Laboratory data on admission for rehabilitation			
Creatinine, mg/dl	1.06 ± 0.48	0.99 ± 0.47	**.01**
Total cholesterol, mg/dl	159.98 ± 38.55	168.40 ± 35.19	**< .01**
Cognitive and functional status on admission for rehabilitation			
Mini-mental status exam	21.0 ± 6.61	21.29 ± 6.91	.47
Cognitive FIM	23.72 ± 6.04	23.67 ± 6.08	.97
Motor FIM	39.22 ± 11.24	40.27 ± 11.50	.29

Statistically significant *p* values are in bold.

The patients with DM had fasting glucose of 128.71 ± 40.8 mg/dl on admission to rehabilitation, and a pre-fracture HbA1c level of 6.6 ± 1.3%. The urine albumin/creatinine ratio among diabetics was 105.62 ± 338.0 mg/g. Abnormal albuminuria was observed in 36.3%; 27.4% had moderately increased albuminuria and 8.9% had severely increased albuminuria (formerly called microalbuminuria and macroalbuminuria, respectively). Among patients with DM, 51.8% received non-insulin anti-diabetic therapies, 31.1% received insulin and 7.3% received a combination of both.

Table 2 describes the rehabilitation outcomes of the patients. All outcomes measured were similar between groups, except for a trend towards increased 1-year mortality among patients with DM (10.9 *vs.* 6.6%, respectively, *p* = .07). Cognitive function did not change during rehabilitation in either group.

**Table 2.** Rehabilitation outcomes of elderly patients with or without diabetes after hip fracture.

Variable	DM	Non-DM	*p* Value
*N* (%)	193 (30.6)	437 (69.4)	
Discharge destination			.32
Home, *n* (%)	178 (92.2)	392 (89.7)	–
Nursing care, *n* (%)	15 (7.8)	45 (10.3)	–
Delta motor FIM	15.6 ± 8.9	14.8 ± 8.8	.35
Recurrent fracture, *n* (%)^a^	13 (6.7)	25 (5.7)	.62
Recurrent hospitalization, *n* (%)^a^	70 (36.3)	142 (32.5)	.35
Mortality, *n* (%)^a^	21 (10.9)	29 (6.6)	.07

^a^Within 1 year of discharge from rehabilitation.

Regression analyses assessing variables that may affect rehabilitation outcomes for the total cohort and for the diabetic patients are presented in Tables 3 and 4, respectively. For the total sample, younger age, male sex, and higher BMI were associated with higher delta motor FIM. None of the clinical indices affected discharge destination or recurrent hospitalizations. Age had a negative effect on recurrent fractures (OR = 0.93, 95% CI [0.88, 0.98], *p* < .01). DM did not have a deleterious effect on rehabilitation outcomes other than on 1-year mortality (OR = 2.78, 95% CI [1.28, 6.04], *p* = .01). Among the patients with DM, higher creatinine levels were associated with additional fractures and hospitalizations at 1-year, while lower BMI and higher total cholesterol were associated with discharge to a nursing care facility.

**Table 3.** Regression analyses of multivariate models predicting rehabilitation outcomes of elderly patients after hip fracture.

Variable	Delta motor FIM^α^	Discharge destination^β^	Recurrent fractures^β^	Recurrent hospitalization^β^	Mortality^β^
Age	−0.18**	1.04	0.93**	1.01	1.11**
Sex (male)	0.10*	1.26	0.82	0.95	0.94
BMI	0.10*	0.93	0.97	1.01	0.87**
Ischaemic heart disease	0.00	0.54	1.72	1.31	0.55
Cerebrovascular disease	**−**0.05	1.43	1.51	1.21	1.30
Chronic kidney disease	**−**0.04	0.59	0.52	1.09	0.27*
Creatinine	0.03	0.83	1.42	1.18	1.93
Cholesterol	**−**0.04	0.99	0.99	0.99	0.99
Diabetes mellitus	0.01	0.95	1.04	1.13	2.78*
*R*^2^ (%)	8.5	5.4	6.7	1.8	14.1

^α^Standardized coefficients of regression predicting delta motor FIM.

^β^Odds ratio of logistic regressions predicting discharge to a nursing care facility at the end of rehabilitation and recurrent fracture, recurrent hospitalization and mortality within 1 year of discharge from rehabilitation.

Statistically significant *p* values were bolded. *p < .05 and **p < .01.

**Table 4.** Logistic regressions of multivariate models predicting rehabilitation outcomes of elderly patients with diabetes mellitus after hip fracture.

Variable	Delta motor FIM^α^	Discharge destination^β^	Recurrent fractures^β^	Recurrent hospitalization^β^	Mortality^β^
Age	−0.26**	1.02	0.89*	1.01	1.01
Sex (male)	0.08	5.25	0.41	1.01	0.74
BMI	0.03	0.76*	0.99	1.02	0.91
Ischaemic heart disease	**−**0.04	0.33	3.21	1.22	1.03
Cerebrovascular disease	**−**0.04	2.15	1.10	1.34	1.03
Chronic kidney disease	**−**0.01	2.77	0.14	0.53	0.37
Creatinine	**−**0.06	0.07	3.18*	3.68*	2.64
Cholesterol	**−**0.03	1.03**	0.99	0.99	1.00
*R*^2^ (%)	4.0	34.5	21.8	5.7	10.9

^α^Standardized coefficients of regression predicting delta motor FIM.

^β^Odds ratio of logistic regressions predicting discharge to a nursing care facility at the end of rehabilitation and recurrent fracture, recurrent hospitalization, and mortality within 1 year of discharge from rehabilitation.

Statistically significant *p* values were bolded. *p < .05 and **p < .01.

## Discussion

In this study, we compared the effectiveness of inpatient geriatric rehabilitation in achieving functional improvement among elderly patients with and without DM after hip fracture. Prediction of rehabilitation outcomes after hip fracture is important, as it may assist in allocating resources more effectively and help coordinate caregivers’ and patients’ expectations.

We found that patients with well-controlled type 2 DM had similar rehabilitation potential compared with non-diabetics, including short-term (delta motor FIM and discharge destination) and long-term (recurrent fractures and hospitalizations) outcomes. These results are encouraging, considering that significantly more co-morbidities were observed in the group with DM. This issue is also important because the incidence of DM is increasing and risk of falls and fragility fractures is higher due to diabetes complications, such as neuropathy and retinopathy.

Studies addressing the effect of DM on post-hip fracture rehabilitation outcomes have presented conflicting results. Lieberman et al. showed no significant effect of DM on post-hip fracture rehabilitation outcomes [21]. However, another prospective study published by the same group concluded that rehabilitation outcomes of diabetic patients are significantly worse. Nevertheless, they suggest that the effect of DM on rehabilitation outcomes is indirect and is manifested through a lower pre-event functional state and higher rate of prior stroke among DM patients [22]. A retrospective study by Huang et al. found that DM had a negative impact on rehabilitation outcomes in terms of lower odds of recovering the ability to walk. However, this study used the Chinese Barthel Index to measure rehabilitation outcomes; composed of ADL and the ability to walk. In addition, the study population was composed of elderly Taiwanese with unique ethnic characteristics [23].

In agreement with our study results, Mizrahi et al. found no difference in rehabilitation outcomes of patients with or without DM. They also demonstrated similar baseline cognitive and motor function between the two groups [17].

Several differences were noted between patients with or without DM in the current cohort. First, diabetic patients were younger than those without DM, as seen in other studies [17,22,23]. This finding might be explained by accelerated osteoporosis and earlier occurrence of hip fractures among patients with DM [24]. The younger age of the diabetic patients may explain how they performed equally well, despite higher prevalence of co-morbidities. Second, patients with DM had higher BMI, which in turn was associated with better short-term (delta motor-FIM) and long-term (1-year mortality) outcomes. This finding is in concert with previous studies that showed better survival among overweight elderly patients [25,26]. Third, among diabetic patients, higher creatinine levels were associated with a more than 3-fold increase in the risk for recurrent fracture within a year. This may be explained by known correlations between renal dysfunction and bone fragility [27], and should guide future studies to explore specific interventions in this very high-risk group of patients.

The diabetic population in our study had several interesting characteristics. All the diabetic patients were classified as type 2. HbA1c levels were rather low (6.6 ± 1.25%). Well-controlled DM is typical in elderly populations. For example, a retrospective cohort of 71,092 patients above age 60, had a mean HbA1c of 7 ± 1.2% [28]. According to the national programme for Quality of Care in the Community in Israel, only 6.9% of elderly patients with DM were poorly controlled (i.e. HbA1C > 9%) [29]. Despite the low HbA1c levels, almost 90% of the patients with DM in our cohort received anti-diabetic therapy: 51.8% non-insulin therapies, 31.1% insulin and 7.3% a combination of both. This raises the suspicion of over-treatment, with presumably higher risk of important side-effects, especially hypoglycaemia, which in turn may result in higher risk of falls and fractures. A large retrospective study of 652,901 elderly men with DM described a J-shaped association curve between HbA1c and the risk of hip fracture. In this cohort, HbA1c < 6.5% was a risk factor for any clinical fracture and hip fracture, especially when combined with insulin therapy [30].

Although the rehabilitation potential between groups was similar, there was a trend towards increased 1-year mortality following rehabilitation among diabetics (10.9 *vs*. 6.6%, respectively, *p* = .07). A multivariate logistic regression showed that DM is a significant risk factor for mortality (OR = 2.78, 95% CI [1.28, 6.04], *p* = .01). These results are in concert with previous studies that showed even higher 1-year mortality rates among diabetics after hip fracture (22.6 *vs.* 10.3%, respectively [23] and 32 *vs.* 12.7%, respectively [11]), which is probably related to higher prevalence of major co-morbidities. Unfortunately, cause of death in the present cohort was not available for analysis.

### Study limitations

Although our study analysed post-hip fracture outcomes of a large cohort of diabetic and non-diabetic patients, it has some limitations. The population was comprised patients hospitalized in a single geriatric rehabilitation department, which may weaken the generalizability of the results. Second, the diabetic patients included in the cohort had surprisingly good diabetes control, potentially leading to better rehabilitation outcomes than might occur in patients with uncontrolled DM. Third, the pre-fracture functional level of each patient was not assessed. Lastly, this was a retrospective study with inherent limitations, such as missing or incorrect data that might bias the results; although, we conducted a thorough review of all medical charts to minimize such biases.

## Conclusions

Elderly patients with well-controlled type 2 DM after hip fracture can achieve similar functional improvements as those without DM, despite significantly more co-morbidities. These results support including patients with DM in post-hip fracture rehabilitation programmes. Nevertheless, this group requires closer post-rehabilitation monitoring and risk-factor control due to a tendency towards higher mortality.

## Data Availability

Data are available upon reasonable request from the corresponding author.

## Previous presentations

The abstract was submitted to 2021 European Congress of Endocrinology (May 2021).

## Appendix 1. Professional interventions of the multidisciplinary rehabilitation team

**Table:**

Discipline	Intervention	Availability
Medical care^a^	Assess and treat health conditions Periodic geriatric assessment	24 h/d, 7 d/weeks
Nursing	Assess physical condition Wound care and manage medications Evaluate self-care skills Evaluate family and home care factors Self-care training Patient and family education	24 h/d, 7 d/weeks
Physical therapy	Assess range of motion and strength Assess gait and mobility Exercise training	5 h/d, 5 d/weeks
Occupational therapy	Evaluate activities of daily living Evaluate cognition and mood Self-care skills training Fabricate splints Treat upper extremity deficits	5 h/d, 5 d/weeks
Speech therapy	Assess and treat speech impairments Assess and treat swallowing impairments	As needed
Nutrition	Assess nutritional status Revise diet to maximize nutrition	5 h/d, 5 d/weeks
Social work	Evaluate family and home care factors Assess psychosocial factors Counselling Liaison with the community	8 h/d, 5 d/weeks

^a^The physician staff includes geriatric specialists available 8 h a day, 5 d a week and general practitioners who attend to urgent medical conditions on evenings and weekends.

## Appendix 2. The functional independence measure (FIM)

**Table:**

Motor subscale	Cognition subscale
Eating	Comprehension
Grooming	Expression
Bathing	Social interaction
Dressing, upper body	Problem solving
Dressing, lower body	Memory
Toileting	**–**
Bladder management	**–**
Bowel management	**–**
Transfers – bed/chair/wheelchair	**–**
Transfers – toilet	**–**
Transfers – bath/shower	**–**
Walk/wheelchair	**–**
Stairs	**–**

Each item is scored on a 7-point ordinal scale, ranging from 1 to 7. The higher the score, the more independent the patient is in performing the task associated with that item. 1 – Total assistance with helper, 2 – Maximal assistance with helper, 3 – Moderate assistance with helper, 4 – Minimal assistance with helper, 5 – Supervision or setup with helper, 6 – Modified independence with no helper, and 7 – Complete independence with no helper.

The total score for the FIM motor subscale (the sum of the individual motor subscale items) is a value between 13 and 91. The total score for the FIM cognition subscale (the sum of the individual cognition subscale items) is a value between 5 and 35. The total score for the FIM instrument (the sum of the motor and cognition subscale scores) will be between 18 and 126.

## Abbreviations

DM: Diabetes Mellitus
FIM: Functional Independence Measure
ROM: Range of Motion
BMI: Body Mass Index;
MMSE: Mini Mental State Examination
